# Toe clearance and velocity profiles of young and elderly during walking on sloped surfaces

**DOI:** 10.1186/1743-0003-7-18

**Published:** 2010-04-28

**Authors:** Ahsan H Khandoker, Kate Lynch, Chandan K Karmakar, Rezaul K Begg, Marimuthu Palaniswami

**Affiliations:** 1 Department of Electrical & Electronic Engineering, The University of Melbourne, Melbourne, VIC 3010, Australia; 2 Biomechanics Unit, Center for Ageing, Rehabilitation, Exercise and Sport, Victoria University, Melbourne, VIC 8001, Australia

## Abstract

**Background:**

Most falls in older adults are reported during locomotion and tripping has been identified as a major cause of falls. Challenging environments (e.g., walking on slopes) are potential interventions for maintaining balance and gait skills. The aims of this study were: 1) to investigate whether or not distributions of two important gait variables [minimum toe clearance (MTC) and foot velocity at MTC (Vel_MTC_)] and locomotor control strategies are altered during walking on sloped surfaces, and 2) if altered, are they maintained at two groups (young and elderly female groups).

**Methods:**

MTC and Vel_MTC _data during walking on a treadmill at sloped surfaces (+3°, 0° and -3°) were analysed for 9 young (Y) and 8 elderly (E) female subjects.

**Results:**

MTC distributions were found to be positively skewed whereas Vel_MTC _distributions were negatively skewed for both groups on all slopes. Median MTC values increased (Y = 33%, E = 7%) at negative slope but decreased (Y = 25%, E = 15%) while walking on the positive slope surface compared to their MTC values at the flat surface (0°). Analysis of Vel_MTC _distributions also indicated significantly (*p *< 0.05) lower minimum and 25^th ^percentile (*Q1*) values in the elderly at all slopes.

**Conclusion:**

The young displayed a strong positive correlation between MTC median changes and IQR (interquartile range) changes due to walking on both slopes; however, such correlation was weak in the older adults suggesting differences in control strategies being employed to minimize the risk of tripping.

## Background

Locomotor behaviour of walking gait is challenged by environmental factors including sloped surfaces. Lower extremity biomechanics for slope walking in humans have been used to provide insight into neural control strategies for different locomotor tasks [1-4]. Recently control strategies during slope walking were analysed using joint kinematics, kinetics and EMG activity results while walking on both negative and positive slopes [5] which suggested that the central nervous system uses different control strategies to successfully walk on slopes. While the differences in lower extremity biomechanics between slope and level walking have the potential to provide insight into these new control strategies, however, no literature has been presented to describe the control strategies for minimizing the risk of tripping in the elderly during slope walking. It has been well documented in the literature that ageing contributes to altered control mechanism of human locomotor balance, which in turn can influence gait patterns. Most falls in older adults are reported during locomotion. Tripping whilst walking is the most commonly reported cause of falls [6], accounting for 53% of falls in healthy older adults [7]. Additionally, falls in the elderly might be linked to declines in the balance control function due to walking on challenging environments. In our earlier studies [8-11], we have identified minimum toe clearance (MTC) as an important gait parameter associated with trip-related falls in older population in successful negotiation of the environment in which we walk. MTC while walking occurs during the mid-swing phase of the gait cycle, and is defined as the minimum vertical distance between the lowest point under the front part of the shoe/foot and the ground. During this MTC event, the foot travels very close to the walking surface and MTC fluctuation has the potential to cause tripping, especially for unseen obstacles. Foot velocity at MTC has been reported to be at its maximum previously [12], however, there have not been any previous attempts to characterize gait control mechanisms using this measure. Foot velocity at MTC represents an important dynamic measure of the foot at the critical event which potentially determines whether the consequent of a trip would be a fall or not.

We have reported that the changes in MTC central tendency/variability are the possible strategies adopted by elderly individuals to minimize tripping risk during level walking [8-10]. However, so far research on tripping risk and MTC analysis [8-10] has been conducted on flat surfaces (laboratory walkways or treadmills with 0° inclination). Previous investigation [3] has shown the human locomotion pattern to be highly adaptive to varying terrains. As our surroundings are by no means flat and also given the fact that negotiating sloped surfaces is a necessity during our everyday locomotion, analysis into slope walking is one way to understand adaptive gait control mechanism and to further explore the causes of trips and falls. In this research, we hypothesize that walking on sloped surfaces would induce altered balance control strategies to minimize the risk of tripping.

This study, therefore, investigated the profiles of two gait measures [MTC and foot velocity at MTC (Vel_MTC_)] on positive and negative slopes. The purpose of this study was twofold. Firstly, to investigate whether or not distributions of two important gait variables and locomotor control strategies to minimize the risk of tripping are altered during walking on sloped surfaces. Secondly, if these strategies exist, are they maintained at young and elderly female age groups or not.

## Methods

### Subjects and experimental design

Nine healthy young female volunteers (age (yr) = 23.9 ± 1.7) were recruited via responding to volunteer notices within Victoria University. Plus eight healthy elderly female volunteers over the age of 65 years (age (yr) = 69.1 ± 5.12) were recruited from retirement villages, older adults' aqua aerobics classes and advertisements placed in a monthly senior citizens' newspaper. All participants were female and they undertook informed-consent procedures as approved by the Victoria University Human Research Ethics Committee. The study protocol was designed to analyse young and elderly subjects walking on a trimline 7600 motorised treadmill at the gradients of -3°, 0° and +3° for 7 minutes. 3° gradient was selected as most walkway ramps are approximately ± 2.9° [13]. All participants were independent living, led an active lifestyle and had no history of falls in the last 2 years, whilst being free of cardiac, musculoskeletal, or orthopaedic troubles that may affect balance or locomotion. Participants wore their own flat, comfortable shoes suitable for walking. This was to ensure an accurate representation of everyday walking, with a heel no higher than 2.5 cm, to minimize shoe effects on the results. Our study protocol had participants walk at their preferred walking speed (PWS) [14,15], which was kept constant across all three gradients. The ranges (min~max) of PWS (treadmill speed) for the young and elderly subjects were 3.0~4.0 km/h (mean = 3.51 km/h) and 1.7~5.0 km/h (mean = 3.14 km/h) respectively.

### MTC Gait Data

**Figure 1:**
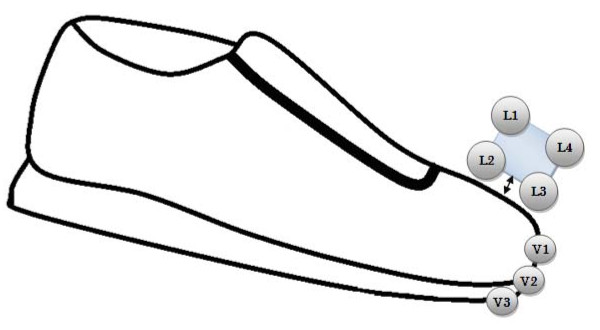
**Optotrak marker positions**. Optotrak marker positions that were used to predict the lowest point on the shoe. L1, L2, L3 and L4 represent the real markers located on rigid body. V1, V2 and V3 (first, third and fifth metatarsals) represent the virtual markers.

Whilst participants walked at the gradients of -3°, 0° and +3°, toe clearance data were collected at 100 Hz using Optotrak Certus motion analysis system (Northern Digital Inc., Canada). Fig. 1 shows marker positions that were used to predict the lowest point on the shoe. The Optotrak system using infrared markers (IRED) and through the construction of rigid bodies and virtual markers, is able to calculate the 3D position of markers (real or virtual) in real time. Northern Digital Inc (NDI) Toolbench was used to construct a rigid body which consists of 4 IRED's (L1, L2, L3 and L4) attached to the shoe. Using the rigid body NDI Toolbench is able to establish the co-ordinates of a point in close proximity, without the presence of a real marker, this point is known as 'virtual marker'. The real markers (IRED) may not accurately represent the lowest point of the shoe. Constructing a virtual marker is achieved through the placement of a tip probe on the desired points (V1, V2, V3; first, third and fifth metatarsals) whereby the computer is able to calculate the location in respect to the origin of the rigid body. Each position location was determined through palpation on the shoe. Toe trajectory and clearance (for each location) over the walking surface for each gait cycle were calculated using a QBasic software program. MTC was determined for each marker location and the minimum of the 3 toe locations (V1, V2 and V3) was used for further analysis. Horizontal velocity at MTC (VelMTC) was then calculated using the central difference method. 2D and 3D accuracy of the motion analysis system used in this study were 0.1 mm and 0.15 mm respectively at 2.25 m distance http://www.ndigital.com/lifesciences/certus-techspecs.php.

### Data Analysis

The total number of gait cycles analysed per subject (i.e., the number of MTC data and hence Vel_MTC _data) varied across the subjects due to their individual preferred walking speed. The range of gait cycles measured was 358 to 567. The extracted MTC and Vel_MTC _data from multiple steps at each gradient was plotted in distribution. Data distribution statistics were determined, including minimum (*min)*, maximum (*max)*, mean(*mean)*, median (*median)*, standard deviation (*STD*), 25^th ^percentile (*Q1*), 75^th ^percentile (*Q3*), interquartile range (*IQR*), upper quartile range (*UQR*), lower quartile range (*LQR*), skewness (*S*) and kurtosis (*K*). *S *> 0 means skew to the right and *K *> 0 means a leptokurtic/peaked distribution. As MTC [8] and Vel_MTC _data were not normally distributed, Kruskal-Wallis non-parametric test (one way analysis of variance) was used to test the effect of age on descriptive statistics of MTC and Vel_MTC _separately at each slope considering PWS as a covariate. Associations between descriptive statistics were determined using Spearman coefficient ρ [16]. Friedman's nonparametric two-way analysis of variance was employed to test the effect of slopes on descriptive statistics of MTC and Vel_MTC _for the two aged groups. To test multi comparisons among three slopes' descriptive statistics, a bonferroni post-hoc test was applied after Friedman test shows significance at *p *< 0.05.

## Results

**Figure 2:**
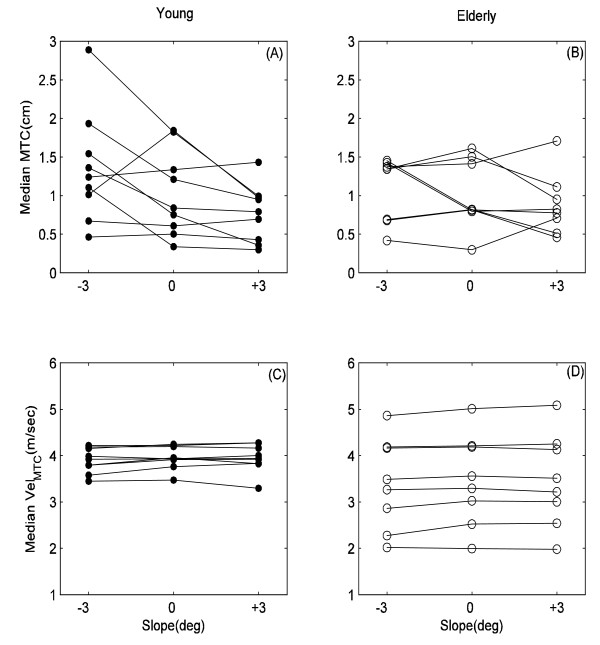
**Median values of MTC and Vel_MTC _at slopes**. Median values of MTC and Vel_MTC _of individual participants during walking at -3°>, 0° and +3° slopes.

Fig. 2 presents *median*MTC and *median*Vel_MTC _for all 17 participants in two groups. It shows there were considerable variation in median values of MTC and Vel_MTC _of individual participants walking at flat as well as sloped surfaces.

### MTC histograms at various slopes

**Figure 3:**
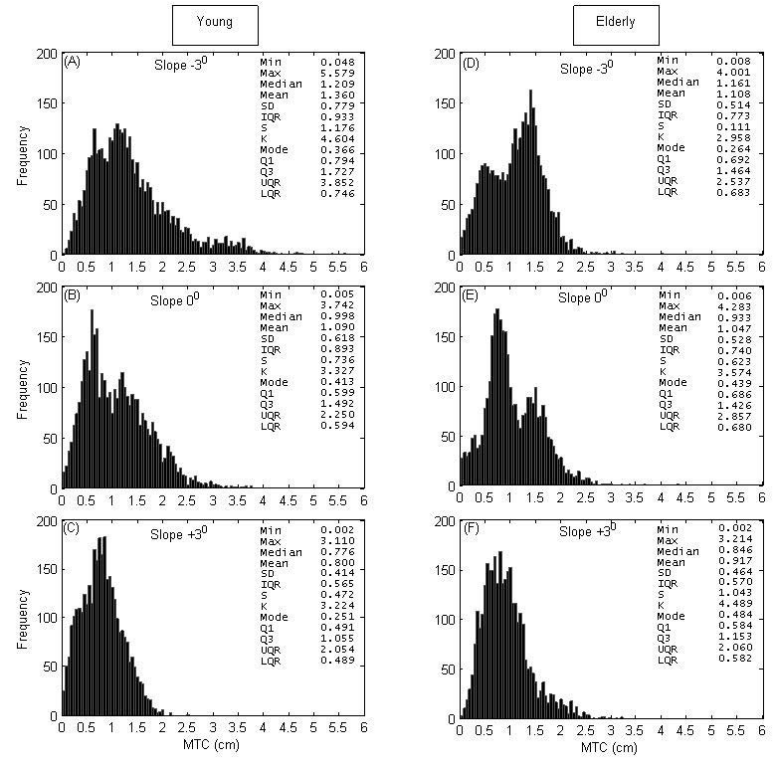
**MTC histograms**. MTC histogram of the young group (left panels) at (A) -3° slope (N = 3714), (B) 0° (N = 3695) and (C) +3° slope (N = 3349) and of the elderly group (right panels) at (D) -3° slope (N = 3313), (E) 0° (N = 3243) and (F) +3° slope (N = 3211). N = number of samples.

Fig. 3 shows MTC histograms for young and elderly groups during walking on various sloped surfaces. These plots reveal some obvious qualitative differences between two groups such as differences in variability and central tendency of MTC. Descriptive statistics of MTC and Vel_MTC _while walking at positive, flat and negative slopes for young and elderly groups are presented in Table 1. Friedman's nonparametric two-way analysis of variance test results show that *max*MTC, *mean*MTC, *STD*MTC and *LQR*MTC at -3° slope were significantly higher than that at +3° in the young group.

**Table 1:** MTC and Vel_MTC _values at slopes

		Young (N = 9) mean (SD)		Elderly (N = 8) mean (SD)		*p *values	
	**Slope(**°)	MTC	Velocity	MTC	Velocity	MTC	Velocity
**Min**	-3	0.39 (0.28)	3.55 (0.28)	0.39 (0.31)	2.84 (1.03)	1.00	0.04*
	0	0.33 (0.25)	3.52 (0.34)	0.35 (0.30)	3.05 (1.05)	1.00	0.04*
	+3	0.27 (0.29)	3.61 (0.33)	0.23 (0.23)	3.06 (1.13)	1.00	0.03*

**Max**	-3	2.96 (1.23)^b^	4.15 (0.27)^a^	2.85 (0.55)	3.72 (0.92)	0.84	0.13
	0	2.43 (0.75)	4.28 (0.23)^a^	2.84 (0.74)	3.83 (0.87)	0.83	0.16
	+3	1.93 (0.66)^b^	4.28 (0.28)	2.50 (0.69)	3.84 (0.91)	0.89	0.18

**Median**	-3	1.36 (0.72)^b^	3.90 (0.27)	1.09 (0.42)	3.39 (0.99)	1.00	0.04*
	0	1.03 (0.55)	3.96 (0.25)	1.01 (0.45)	3.47 (0.98)	1.00	0.06
	+3	0.77 (0.37)^b^	3.95 (0.30)	0.88 (0.40)	3.47 (1.00)	1.00	0.08

**Mean**	-3	1.37 (0.71)^b^	3.90 (0.27)	1.13 (0.39)	3.39 (0.99)	1.00	0.04*
	0	1.06 (0.56)	3.96 (0.25)	1.06 (0.44)	3.48 (0.98)	1.00	0.06
	+3	0.79 (0.36)^b^	3.95 (0.30)	0.91 (0.38)	3.47 (1.00)	1.00	0.07

**STD**	-3	0.38 (0.16)^b^	0.09 (0.01)	0.34 (0.08)	0.12 (0.03)	1.00	1.00
	0	0.31 (0.11)	0.10 (0.02)	0.32 (0.12)	0.11 (0.02)	1.00	1.00
	+3	0.24 (0.06)^b^	0.10 (0.02)	0.31 (0.08)	0.11 (0.03)	1.00	1.00

**IQR**	-3	0.48 (0.25)	0.12 (0.02)	0.42 (0.11)	0.14 (0.04)	1.00	1.00
	0	0.37 (0.15)	0.12 (0.02)	0.36 (0.14)	0.14 (0.03)	1.00	1.00
	+3	0.30 (0.05)	0.13 (0.02)	0.37 (0.12)	0.14 (0.03)	1.00	1.00

**Skewness**	-3	0.72 (0.70)	-0.31 (0.43)	0.94 (0.53)	-0.58 (0.73)	0.99	1.00
	0	0.89 (0.64)	-0.34 (0.29)	1.33 (0.85)	-0.15 (0.35)	0.98	1.00
	+3	0.83 (0.45)	-0.04 (0.26)	1.14 (1.07)	-0.06 (0.39)	0.99	1.00

**Kurtosis**	-3	5.33 (3.05)	4.09 (1.67)	5.44 (2.00)	5.81 (3.60)	0.70	0.30
	0	5.54 (2.46)	4.60 (1.27)	8.66 (6.38)	3.90 (1.16)	0.86	0.39
	+3	5.04 (1.90)	3.54 (0.61)	8.72 (8.40)	4.10 (1.34)	0.53	0.87

**Mode**	-3	0.94 (0.70)	3.84 (0.33)	0.86 (0.39)	3.35 (1.05)	1.00	0.05*
	0	0.71 (0.70)	3.79 (0.33)	0.92 (0.39)	3.46 (0.92)	1.00	0.06
	+3	0.56 (0.37)	3.85 (0.40)	0.73 (0.48)	3.33 (1.16)	1.00	0.05*

**Q1**	-3	1.11 (0.61)	3.84 (0.27)	0.89 (0.40)	3.32 (1.00)	1.00	0.04*
	0	0.86 (0.51)	3.90 (0.25)	0.84 (0.42)	3.41 (0.99)	1.00	0.04*
	+3	0.63 (0.35)	3.88 (0.31)	0.70 (0.36)	3.40 (1.02)	1.00	0.04*

**Q3**	-3	1.59 (0.84)	3.96 (0.26)	1.32 (0.41)	3.46 (0.97)	1.00	0.06
	0	1.23 (0.61)	4.02 (0.25)	1.21 (0.48)	3.55 (0.97)	1.00	0.08
	+3	0.92 (0.37)	4.01 (0.29)	1.07 (0.43)	3.54 (0.99)	1.00	0.09

**UQR**	-3	1.37 (0.59)	0.19 (0.05)	1.54 (0.63)	0.26 (0.08)	1.00	1.00
	0	1.21 (0.42)	0.27 (0.05)	1.63 (0.75)	0.28 (0.10)	0.99	1.00
	+3	1.00 (0.45)	0.27 (0.14)	1.43 (0.70)	0.31 (0.17)	0.99	1.00

**LQR**	-3	0.72 (0.39)^b^	0.30 (0.11)	0.50 (0.16)	0.48 (0.20)	1.00	1.00
	0	0.53 (0.27)	0.38 (0.11)	0.49 (0.19)	0.36 (0.13)	1.00	1.00
	+3	0.36 (0.14)^b^	0.27 (0.07)	0.47 (0.15)	0.34 (0.14)	1.00	1.00

Descriptive statistics of MTC and Vel_MTC _of the young and elderly group walking at -3°, 0°, +3° slopes. ^a^significantly different between -3° and 0° at *p *< 0.01. ^b^significantly different between -3° and +3° at *p *< 0.01. *significant ageing effect at *p *< 0.05.

### Vel_MTC _histograms at various slopes

**Figure 4:**
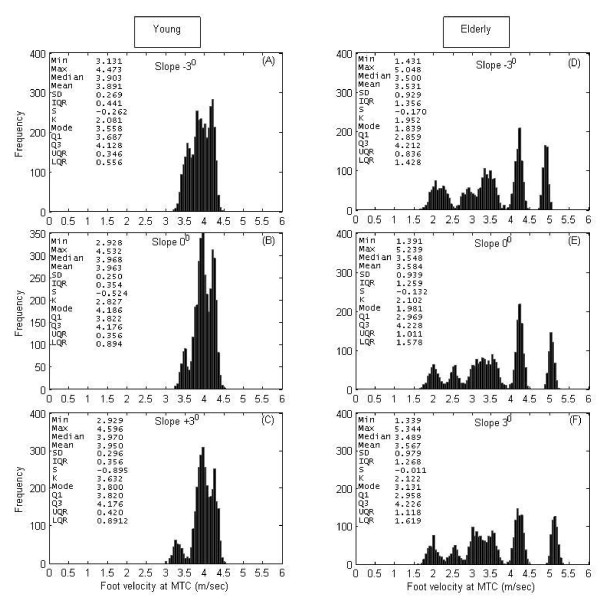
**Vel_MTC _histograms**. Foot velocity at MTC (Vel_MTC_) histogram of the young group (left panels) at (A) -3° slope (N = 3714), (B) 0° (N = 3713) and (C) +3° slope (N = 3379), and of the elderly group (right panels) at (D) -3° slope (N = 3340), (E) 0° (N = 3283) and (F) +3° slope (N = 3215). N = number of samples.

Fig. 4 shows histograms of Vel_MTC _for young and elderly populations during walking on various sloped surfaces. According to the results presented in Table 1, *median*Vel_MTC _of both groups was found to be decreased (1.5% for young group; 2.3% for elderly group) at negative slope but remained relatively unchanged at positive slope. *IQR *and *STD *of Vel_MTC _in both groups remained unchanged at slopes. However, *max*Vel_MTC _at -3° slope in young group was found to be significantly lower than that at flat(0°) surface (Table 1). *Q1*Vel_MTC _and *min*Vel_MTC _at all conditions (+3°, 0° and -3°) were found be significantly (*p *< 0.05) lower in the elderly group. Also, central tendency measures (*mean*Vel_MTC_, *median*Vel_MTC _and *mode*Vel_MTC_) of Vel_MTC _in the elderly group walking at negative slope(-3°) were found to be significantly (*p *≤ 0.05) lower than that of the young group. No significant differences between groups were found for other measures. However, it is interesting to note that distributions of MTC and Vel_MTC _in both groups are oppositely skewed (Table 1).

### Relationships between the changes of medianMTC and IQRMTC due to changes of slopes

**Figure 5:**
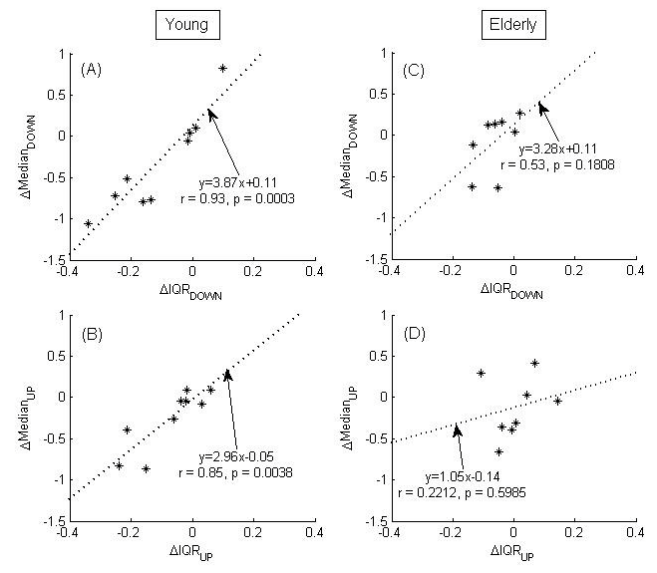
**Median-IQR strategies**. Relationship between ΔMedian and ΔIQR of MTC for young adults (left panels A, B) and elderly adults (right panels C, D) during the change of slopes from 0° to +3° (up) and slopes from 0° to -3° (down). Significant correlation (*p *< 0.01) was found in the young group. ρ = Correlation coefficient. (See text for details).

Fig. 5 shows that walking on both slope changes in downward (from 0° to -3°) (panel A) and upward (from 0° to +3°) (panel B) induced significant correlations (ρ = 0.93, *p *= 0.0003; ρ = 0.85, *p *= 0.0038) between changes of *median*MTC and *IQR*MTC for the young adults. In comparison, there are no such significant relationships found in the elderly adults due to changes in slopes (panel C and D).

### Correlations among the measures of MTC and Vel_MTC_

Table 2 summarize the correlations among (*media*n, *IQR) of *MTC and (*median*, *IQR*) of Vel_MTC _respectively within each age group. There were significant (*p = <*0.01) positive relationship of *IQR*Vel_MTC _with *median*MTC(ρ = 0.83) and *IQR*MTC(ρ = 0.81) in the young adults while walking at 0° slope (Table 2b). However, no such significant relationships were found in the same group while walking at sloped surfaces (-3° and +3°) (Table 2a, c).

**Table 2:** Correlations among Median-IQR of MTC and Vel_MTC _values at slopes

		*Median Vel*_MTC_		*IQR Vel*_MTC_	
		
		Young	Elderly	Young	Elderly
***(a) Slope -3***°	***Median *MTC**	0.13(0.61)	-0.25(0.14)	0.43(0.23)	0.31(0.83)
	
	***IQR *MTC**	0.15(0.79)	0.13(0.46)	0.20(0.69)	-0.13(0.46)

***(b) Slope 0***°	***Median *MTC**	0.02(0.80)	-0.49(0.34)	0.89(0.01)*	0.46(0.08)
	
	***IQR *MTC**	0.02(0.86)	-0.28(0.67)	0.86(0.01)*	0.20(0.61)

***(c) Slope +3***°	***Median *MTC**	0.03(0.90)	-0.15(0.78)	0.25(0.33)	0.43(0.25)
	
	***IQR *MTC**	0.29(0.48)	0.06(0.78)	-0.36(0.64)	0.16(0.25)

Spearman correlations, ρ (*p *value) among median and IQR of MTC and Vel_MTC _for the young and elderly group walking at (a) -3°, (b) 0° and (c) +3° slopes. *significance at *p *< 0.01.

In the elderly group, in contrast, no significant relationships among descriptive statistics of MTC and *median*Vel_MTC_, *IQR*Vel_MTC _were found (Table 2).

## Discussion

The results of this study highlight the implications of two gait variables, i.e. MTC and foot velocity at MTC (Vel_MTC_) that have been utilized to characterize gait patterns of the young and elderly subjects during walking at various slopes. The trajectory of the foot during gait is a precise end-point control task. MTC has close linkage with tripping risks during walking and its characteristics have been used to effectively recognize trip related fallers from non-fallers [9,15]. Furthermore, foot velocity at MTC represents an important dynamic measure of the foot at the critical event which potentially determines whether the consequent of a trip would be a fall or not. For example, foot moving with a high horizontal velocity is more likely to result in a fall following contact with an over ground obstacle or obstruction. Horizontal foot velocity at MTC has been reported to be at its maximum previously [12], however, there have not been any previous attempts to characterize gait control mechanisms using this measure.

### Strategic relationships

#### a) Strategies employed at flat surface (0°)

Begg [8] demonstrated that MTC distribution statistics could provide insight into possible strategies employed by individuals to exert control on the foot at MTC walking on flat surface (0°). Out of the strategies, the simplest and most effective one is the Median-IQR strategy i.e., to reduce the variability if the foot comes very close to the ground. The results from the present study support the possible strategies suggested by the previous study. For example, during flat surface walking, although statistically not significant, *median*MTC and *IQR*MTC were both lower for the elderly group compared to young group. This suggest that the elderly could have applied increased control by lowering variability (i.e. less IQR) due to their lower MTC height over the ground to avoid potential tripping risk [8].

#### b) Strategies employed at negative slope (-3°)

At negative slope, young group demonstrated positive correlation of change in *median*MTC with the change in *IQR*MTC (in Fig. 5A). Although intra-subject variation can be noticed, but the overall strategic measure of strong positive correlation of *median*MTC with *IQR*MTC was significant (ρ = 0.93, *p *= 0.0003) for this group. The elderly group also demonstrated (in Fig. 5C) a positive correlation of *median*MTC with *IQR*MTC, however, that relation was not significant (*p *= 0.1808). It appears that the Median-IQR control strategy employed to reduce tripping risk is being disturbed or broken down in the older adults while walking on the negative gradient terrain although the young ones are able to still maintain this strategy as a group.

#### c) Strategies employed at positive slope (+3°)

At positive slope, as similar to the strategies employed at negative slope, young group demonstrated strong and significant (ρ = 0.85, *p *= 0.0038) positive relation of change in *median*MTC with the change in *IQR*MTC (in Fig. 5B). Similarly, the elderly group demonstrated (in Fig. 5D) a positive but weak (ρ = 0.22, *p *= 0.5985) correlation. These results indicate that the strategies adopted by elderly group in reacting to the challenges of positive slope walking might be individual specific rather than the unique Median-IQR strategy for everyone. It will be interesting to investigate what, if any, other strategy is being switched on by the individual elderly participants under such condition. All in all, these age-related changes in MTC statistics suggest that presence or absence of strategies employed by the elderly group are different to those employed by the young group which might have implications for increased tripping incidences during walking on sloped surfaces.

### Ageing effects

#### a) Walking on sloped surfaces for the Young and elderly

Although statistically not significant, possibly due to the small sample size used in this research, *median*MTC and *median*Vel_MTC_of young group (i.e. 1.03 cm and 3.96 m/sec) were higher than that of elderly group (i.e., 1.01 cm and 3.47 m/sec) at level walking. Slower foot velocity might be a safety mechanism adopted by the elderly. Winter [12] reported lower mean MTC values in the elderly than that in the young and also found no significant difference between two aged groups. Histograms of Vel_MTC _for young and elderly populations during walking on sloped surfaces revealed that there were potentially 5 sub-groups within the elderly group and 3 sub-groups within the young group (see Fig. 4). Important information could be lost in group-based analysis and only an individual-based approach might show the different strategies employed and which individuals are at a greater risk of tripping. For example, some elderly subjects shown in Fig. 2 might be at a higher risk than others because of their lower *median*MTC and higher *median*Vel_MTC_. The group histograms also indicate that there might be potentially 5 sub-groups within the elderly group and 3 sub-groups within the young group. This may be due to the sparcity of data - with more subjects, these histograms would not be as multimodal.

Significant differences in *Q1*Vel_MTC _values at all slopes (Table 1) could suggest that only the lower end of Vel_MTC _distribution is affected due to ageing. Therefore, *min*Vel_MTC _were also found to be significantly different between the two aged groups at all slopes. Positively skewed MTC and negatively skewed Vel_MTC _at level and sloped walking (Table 1) could be common safety mechanism adopted by both groups. Reduction in mean and median Vel_MTC _at negative slope in both groups are evident but significantly lower Vel_MTC _in the elderly group compared to the young group might indicate slower Vel_MTC _as an additional safety mechanism adopted by the elderly group while walking on negative slope surfaces.

The preferred walking speed (PWS) adopted by both groups in this study were slower than those reported in the literature (e.g., [17]). One possible reason for this difference could be that the participants might have deliberately selected a slower PWS at 0° slope, because they thought they would be required to maintain the same PWS on both positive and negative slopes.

#### b) Combined strategies using measures of MTC and Vel_MTC_

At level walking, *IQR*Vel_MTC _(i.e. Vel_MTC _variability) in young adults maintains positive correlations with *median *and *IQR *of MTC (Table 2). This could be other strategies employed by the young adults at level walking because such correlations were absent at sloped walking. On the other hand, elderly adults did not show such relationships. These observations need to be investigated in more details with a larger sample size.

## Conclusion

The usefulness of MTC and foot velocity at MTC analysis for characterizing the gait patterns at both positive and negative slopes was explored in this study. The findings of this study suggest altered distribution of MTC and foot velocity at MTC as well as different control strategies employed by the young and elderly adults to minimize tripping risk due to walking on sloped surfaces. The young adults displayed a strong positive correlation between MTC median changes and IQR changes due to walking on both slopes; however, such correlation was weak in the older adults suggesting differences in control strategies being employed to minimize the risk of tripping. These results need to be further explored in a larger sample size as well as in other population. A better understanding of this fundamental adaptive gait control information could be useful in the design of future gait diagnostic or screening tools. The results of this study can also be used to understand the normative requirements of descending or ascending ramps during natural walking leading to another potential application area.

## Competing interests

The authors declare that they have no competing interests.

## Authors' contributions

AHK, KL, CKK and MP conceived the study, evaluated the data, performed data analyses and wrote the manuscript. RKB and KL recruited subjects, managed data acquisition and participated to drafting of the manuscript. All authors read and approved the final manuscript.
